# Supplementary material for: Network segregation is associated with processing speed in the cognitively healthy oldest-old
Source: eLife. 2025 Mar 26;14:e78076. doi: 10.7554/eLife.78076 (PMC12097785; doi:10.7554/eLife.78076)
Supplement: Supplementary file 4. [file elife-78076-supp4.docx]

**Supplementary File 4**

|  | **Memory** | **Working Memory** | **Language** | **Executive Functioning** |
| --- | --- | --- | --- | --- |
| FPN Segregation | r=.073  p=.382 | r=.124  p=.135 | r=.06  p=.473 | r=.167  p=.044 |
| DMN Segregation | r=-.017  p=.84 | r=.092  p=.729 | r=.137  p=.098 | r=.108  p=.192 |
| CON Segregation | r=-.102  p=.218 | r=.008  p=.921 | r=-.013  p=.869 | r=.08  p=.337 |

**Correlations between Cognitive Domains and Network Segregation:** results of a Pearson correlation between domain factor scores for each cognitive domain vs segregation of the three networks.
